# TMS-Induced Cortical Potentiation during Wakefulness Locally Increases Slow Wave Activity during Sleep

**DOI:** 10.1371/journal.pone.0000276

**Published:** 2007-03-07

**Authors:** Reto Huber, Steve K. Esser, Fabio Ferrarelli, Marcello Massimini, Michael J. Peterson, Giulio Tononi

**Affiliations:** Department of Psychiatry, University of Wisconsin, Madison, Madison, Wisconsin, United States of America; University of California, Irvine, United States of America

## Abstract

**Background:**

Sleep slow wave activity (SWA) is thought to reflect sleep need, increasing in proportion to the length of prior wakefulness and decreasing during sleep. However, the process responsible for SWA regulation is not known. We showed recently that SWA increases locally after a learning task involving a circumscribed brain region, suggesting that SWA may reflect plastic changes triggered by learning.

**Methodology/Principal Findings:**

To test this hypothesis directly, we used transcranial magnetic stimulation (TMS) in conjunction with high-density EEG in humans. We show that 5-Hz TMS applied to motor cortex induces a localized potentiation of TMS-evoked cortical EEG responses. We then show that, in the sleep episode following 5-Hz TMS, SWA increases markedly (+39.1±17.4%, p<0.01, n = 10). Electrode coregistration with magnetic resonance images localized the increase in SWA to the same premotor site as the maximum TMS-induced potentiation during wakefulness. Moreover, the magnitude of potentiation during wakefulness predicts the local increase in SWA during sleep.

**Conclusions/Significance:**

These results provide direct evidence for a link between plastic changes and the local regulation of sleep need.

## Introduction

During non rapid eye movement (NREM) sleep the EEG is dominated by slow waves of high amplitude, which are generated by millions of neurons switching from a depolarized up-state to a hyperpolarized down-state [1], [2]. As shown by studies in many species, the amount of sleep slow waves, usually measured as slow wave activity (SWA), EEG power in the 1–4.5 Hz range, is homeostatically regulated [3]: specifically, SWA increases with the duration of previous wakefulness and declines exponentially during sleep. Thus, SWA provides a reliable indicator of sleep pressure and may be linked to the restorative function of sleep [4], [5]. However, the neural mechanisms underlying the increase in SWA with increasing sleep pressure remain unknown.

An important advance has come from recent work showing that sleep SWA can be regulated locally in the cerebral cortex [6]–[8]. Intriguingly, some of this work points to a link between local SWA regulation and synaptic plasticity [6], [8]. Thus, in a study with high-density (hd)-EEG in humans, sleep SWA was locally increased after a visuomotor learning task involving right parietal cortex, but not after a kinematically equivalent motor task that did not require learning [6]. In a second study, sleep SWA was locally decreased over right sensorimotor cortex if a subject's left arm had been immobilized during the day, leading to a deterioration in motor performance and to a decrease in somatosensory and motor evoked potentials [8]. Altogether, these experiments suggest that sleep SWA is affected by plastic changes in local cortical circuits and, more specifically, that SWA should increase with synaptic potentiation and decrease with synaptic depression [9]. This hypothesis is supported by computer simulations showing that stronger synapses lead to increased SWA by enhancing neuronal synchronization, whereas weaker synapses have the opposite effect [10].

To test this hypothesis directly, it is important to investigate whether established paradigms for inducing synaptic plasticity, such as long-term potentiation (LTP) [11], yield the predicted changes in sleep SWA. In animals *in vivo*, LTP is classically induced by high-frequency electrical stimulation (5–15 Hz) and assessed by recording changes in population responses to test stimuli [11]. In humans, it has recently become possible to approximate this classic protocol non-invasively by combining transcranial magnetic stimulation (TMS) with hd-EEG [12]: high-frequency electrical stimulation can be safely substituted by repetitive TMS (rTMS), while changes in cortical responses to test TMS pulses can be assessed with hd-EEG. Accordingly, in the present study subjects underwent a 5-Hz rTMS potentiation protocol and a sham session, while cortical responses were monitored using hd-EEG. Thereafter subjects went to sleep and local changes in SWA were investigated with sleep hd-EEG. The results show that rTMS but not sham stimulation produces a local potentiation of cortical responses, and that this potentiation is followed as predicted by a local increase in sleep SWA, in line with the hypothesis that sleep regulation is linked to synaptic plasticity.

## Results

### 5-Hz rTMS results in increased motor evoked potentials and TMS-evoked EEG responses

**Figure 1:**
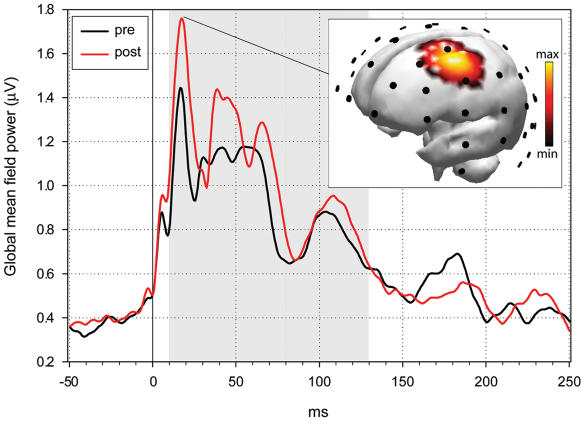
Responses to TMS before and after 5 Hz conditioning. Total activation produced by TMS as measured by the global mean field power (GMFP) derived from all 60 electrodes. The GMFP was increased between 10 and 130 ms post stimulus (gray area) following rTMS (pre, 112.9±37.6 µV; post, 196.1±43.7 µV; p<0.05, paired t-test, n = 10). Inset: Source localization of the average TMS-evoked EEG response at the earliest significant component, 18 ms after magnetic stimulation of the left motor cortex, before the rTMS conditioning (n = 8 subjects). Activity is color coded and projected onto the Montréal Neurological Institute (MNI) standard brain. Electrode positions are indicated by black dots.

TMS was targeted to the hand area of the left motor cortex. Analysis of motor evoked potentials collected immediately before and after the rTMS conditioning confirmed previous studies [13], [14] indicating that motor responses to TMS increase significantly following 5 Hz rTMS (27.0±7.8%; p<0.05, two-tailed unpaired t-test, n = 20 trials, peak-to-peak amplitude of motor evoked potentials). Measuring TMS-evoked EEG responses allowed us to investigate the effect of rTMS conditioning directly on cortical responses. TMS-evoked EEG responses revealed several fast early (10–80 ms) and slower late components (81–200 ms; Figure 1). The earliest apparent peak occurred at 18 ms. Electrode-magnetic resonance (MR) coregistration localized the corresponding maximal current source (see Methods) to left premotor cortex around electrode 9 (Figure 1, inset). A comparison of the TMS-evoked response before and after rTMS conditioning revealed a significant increase in the amplitude of components comprised between 10 and 130 ms (Figure 1, gray area, p<0.05, paired t-test, average global mean field power (GMFP) between 10 and 130 ms).

### Potentiation of TMS-evoked EEG responses is followed by a local increase in SWA during subsequent sleep

**Figure 2:**
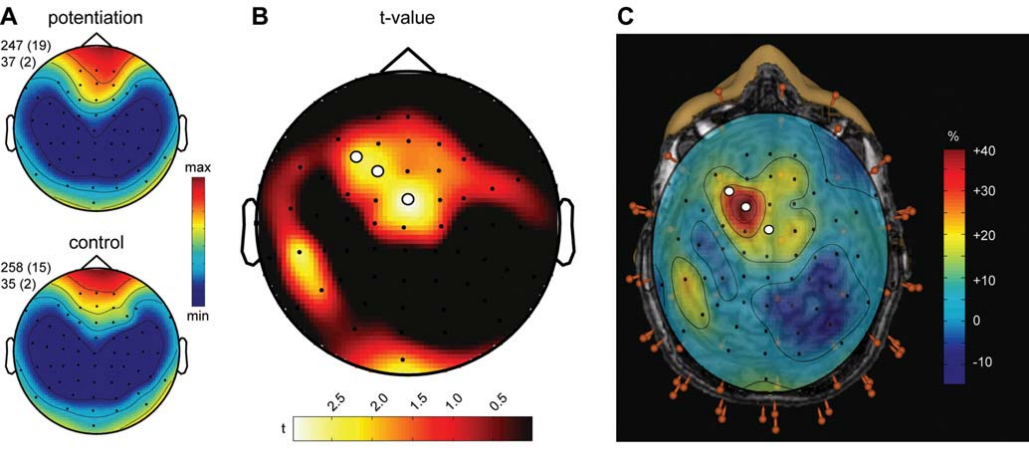
Changes in local SWA homeostasis during sleep after 5-Hz rTMS conditioning. A. Topographic distribution of SWA after 5-Hz conditioning (top) and the sham control (bottom) condition. Average EEG power density at 1–4.5 Hz (n = 10 subjects) for the first 30 minutes of NREM sleep. Values were normalized by total power for the recording, color coded, plotted at the corresponding position on the planar projection of the scalp surface, and interpolated (biharmonic spline) between electrodes (dots). Values to the left of the topographic plots represent maximal and minimal power (in percentage of the overall mean) with standard errors in parenthesis. B. Topographic distribution of the t-value for the comparison between the 5-Hz conditioning and sham control condition (two-tailed paired t-test). White dots indicate electrodes showing significant differences after statistical non-parametric mapping (see methods). C. Anatomical localization of the three electrodes showing a significant difference in SWA during the first 30 min of NREM. All 60 electrodes (red pins) were digitized and co-registered with the subject's magnetic resonance images. When the topographic distribution of the percentage change of SWA after the TMS conditioning compared to the control condition was projected onto the brain, the three significant electrodes projected onto left premotor cortex (white dots).

After the TMS session, the room was darkened and the subjects were allowed to sleep while we recorded their first NREM sleep episode. Subjects showed the usual progression of sleep stages in both the rTMS and the sham TMS session (see Table 1). Sleep onset occurred on average 29±5 min after the end of rTMS conditioning. Average power spectra of consecutive 20-second epochs during the first 30 minutes of NREM sleep showed that SWA was prevalent in anterior regions, in accordance with previous studies [15], [16], and that the topographic pattern of SWA was highly reproducible across nights (Figure 2A). After the rTMS session, compared to the sham session, there was a significant increase of SWA at a cluster of left central electrodes (electrodes 8, 9 and 19; Figure 2B, C). The peak SWA increase occurred at electrode 9 and amounted to 39.1% (±17.4%, p<0.01, two-tailed paired t-test after Statistical non-Parametric Mapping, SnPM). Electrode 9, overlaying left BA 6, was the same electrode at which we observed the maximal current increase in TMS-evoked responses after rTMS conditioning. Thus, rTMS conditioning leaves a local trace in the sleep EEG, and the trace corresponds topographically to the site of increased TMS-evoked EEG response. We found no significant correlation between the time interval from the end of the rTMS conditioning to sleep onset and the local increase of SWA at the beginning of sleep (r = 0.2, p = 0.5). Other frequency ranges, i.e. the spindle frequency range (12–15 Hz), did not show any significant topographical difference (no electrode reached p<0.05 using SnPM, topographical differences are shown in Supplemental Figure S1).

**Table 1:** Sleep architecture for the first NREM sleep episode.

(n = 10)	5-Hz rTMS		Sham control	
	Mean	s.e.m	Mean	s.e.m
Sleep latency (min)	9.2	2.0	7.3	1.1
Total sleep time (min)	53.9	4.0	57.0	4.3
Waking (%)	16.8	4.8	15.4	3.6
Waking after sleep onset (%)	4.8	2.6	7.5	2.2
Stage 1 (%)	9.9	2.1	11.2	3.3
Stage 2 (%)	30.4	5.1	32.2	4.5
Slow-wave sleep (%)	41.2	7.6	38.9	8.0
NREM sleep (%)	71.7	6.3	71.2	6.4
Movement time (%)	1.2	0.3	2.2	0.6

No significant differences were observed between the 5-Hz rTMS and the sham control condition.

**Figure 3:**
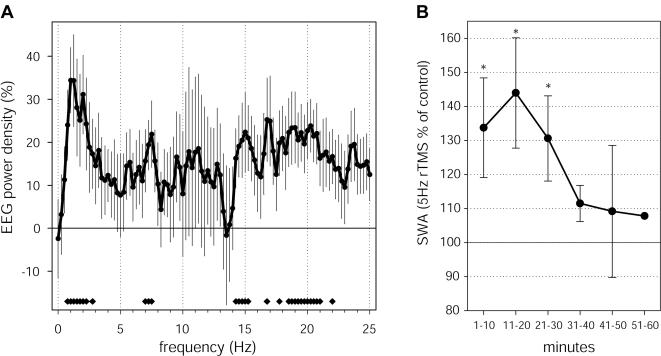
Characteristics of the local SWA change after 5-Hz rTMS conditioning. A. Frequency-specificity of power changes. EEG power density spectrum for the first 30 minutes of NREM sleep. Average power change across subjects for electrode 9, where the peak SWA increase was observed. Values represent the % change after the 5-Hz conditioning with respect to after the sham control condition (mean±SEM for 0.25-Hz bins, n = 10). Bottom bars indicate frequency bins for which power in the 5-Hz conditioning condition differed significantly from the sham control condition (two-tailed paired t-test). B. Time course of SWA changes after 5-Hz conditioning. The change in average EEG power in the 1–4.5 Hz band was calculated for 6 consecutive 10-min intervals during the first NREM sleep episode. As in A, we selected power at electrode 9, corresponding to the peak SWA increase. Stars indicate a significant increase of SWA after the 5-Hz conditioning compared to the sham control condition (p<0.05, two-tailed paired t-test).

In our previous study, visuomotor learning produced a local increase in sleep EEG power mainly in the SWA frequency range, with concomitant increases in the theta range and just above the spindle range (15–16.5 Hz) [6]. As shown in Figure 3A, at electrode 9, where we observed the largest increase in SWA, the spectral changes in sleep EEG power produced by rTMS conditioning were similar to those observed in the learning study, with a peak increase in the frequencies below 3 Hz [6].

We then examined the time course of the relative increase of SWA within the first NREM sleep episode after rTMS conditioning by dividing it into 10 min intervals. We found that the level of SWA remained elevated for the first 30 minutes of NREM sleep (Figure 3B). SWA then decreased progressively in the course of the sleep episode, as revealed by a significant drop from the first 30 to the second 30 minutes of NREM sleep (p<0.05, paired t-test). With the exception of power in the high spindle frequency range no other frequency range showed a similar trend (Supplemental Figure S1). Thus, a local trace of rTMS conditioning, manifested mainly as an increase in SWA, was present for at least the first 30 minutes of sleep, and showed a decreasing trend in the course of the sleep episode.

### The increase in TMS-evoked responses predicts the local increase of sleep SWA

**Figure 4:**
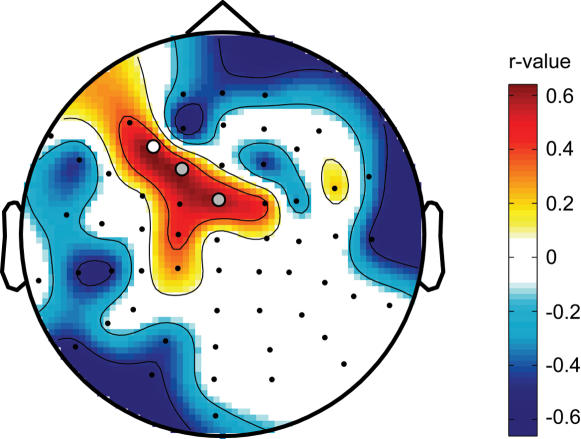
The increase in TMS-evoked responses predicts the local increase of SWA. Topographic depiction of positive correlations between SWA change and the change in global mean field power in the late component of the TMS-evoked response after 5-Hz rTMS conditioning. For each subject the change in activity between 100 and 140 ms before and after the 5-Hz conditioning was calculated and correlated with the change in SWA at each electrode. Filled circles indicate electrodes showing a significant correlation (white circle, r = 0.65, p<0.05 at electrode 8, n = 10), or a trend (grey circles, p<0.1).

Finally, we asked whether the increase of SWA over the left premotor area was predicted by the increase in the amplitude of the TMS-evoked response induced by rTMS conditioning to the left motor cortex. To determine the best predictor of the local increase in SWA after 5-Hz rTMS conditioning, we calculated correlation coefficients for the components of the global mean field power of the TMS-evoked response in relation to the local increase of SWA. We found that the change in amplitude of a late component between 100 and 140 ms was the best predictor of the local increase of SWA (r = 0.65, p<0.05 at electrode 8, n = 10, average GMFP between 100 and 140 ms). Figure 4 illustrates topographically across all electrodes the correlation values between the change in amplitude of the evoked response between 100 and 140 ms and the change in SWA during subsequent sleep. Positive correlations were found for three electrodes just anterior to the site of stimulation, electrodes 8, 9 and 19. The probability that such correlations might occur by chance was <0.0063, which is the threshold for significance after Bonferroni correction for multiple testing.

## Discussion

The results presented here show that high-frequency rTMS conditioning over motor cortex led to a local increase in the amplitude of EEG responses to TMS pulses, indicative of potentiation of premotor circuits, followed during subsequent sleep by a prominent local increase in SWA. Moreover, the potentiation of the EEG response to TMS pulses and the increase in sleep SWA were localized to the same premotor cortical region and were positively correlated. Together, these results indicate that changes in synaptic efficacy lead to changes in local sleep regulation, as reflected by SWA, and thus provide evidence for a tight relationship between synaptic plasticity and sleep need.

### TMS-induced potentiation of cortical responses

In animal studies, LTP is classically induced by high-frequency electrical stimulation, and assessed by recording population responses to individual pulses [11]. A large body of evidence shows that electrically induced LTP is associated with characteristic synaptic changes, such as the increased postsynaptic incorporation of glutamate receptor subunits, that lead to elevated synaptic efficacy [17], [18]. In humans, high-frequency rTMS paradigms are increasingly used to increase the efficacy of targeted circuits [12]–[14], [19]–[22]. The occurrence of long-term potentiation of responses after high-frequency rTMS was initially inferred on the basis of behavioral changes [20], [21] and especially of increased motor evoked potential amplitude to test TMS stimuli [12]–[14], [22]. These findings suggest that 5-Hz conditioning leads to long-term changes in synaptic efficacy. For example, the increased amplitude of motor evoked potentials lasted for at least 40 minutes [12]–[14], [22]. A cortical site of action was suggested by cervical epidural recordings showing an increase in descending motor cortical activity [23]. Furthermore, a study in rats found that rTMS resulted in increased NMDA binding [24], an important requirement for LTP [25].

In this work, we took advantage of the availability of simultaneous TMS/hd-EEG [26], [27] to record directly changes in cortical responses to TMS pulses before and after 5-Hz stimulation (see also [12]). As shown here, the components between 10 and 130 ms of the TMS-evoked EEG responses were significantly increased in amplitude after rTMS, but not after sham stimulation. These results provide a non-invasive, direct demonstration of cortical changes in TMS-evoked response consistent with LTP in humans (see also [12], [28], [29]. Other indications that rTMS can have a persistent influence on cortical function come from neuroimaging studies showing increased glucose metabolism in premotor cortex for at least 10 minutes after 5 Hz stimulation [30]. Another study reported increased sensory discrimination and expanded sensory maps after 5Hz stimulation of somatosensory cortex [20]. The present finding of an increase in sleep SWA for up to an hour after rTMS further indicates that rTMS can leave long-lasting traces, raising the question of when and how such traces disappear. This finding also suggests that the induction of local SWA changes during sleep may be employed to monitor the efficacy of rTMS treatments in brain disorders such as depression [31], and to correlate that efficacy with clinical response.

In the present study, the increased response after 5 Hz TMS to left motor cortex was observed specifically over left premotor cortex. This neighboring area is a main target of projections from motor cortex itself, as indicated by functional imaging in humans [32], and by anatomical [33] and electrophysiological evidence [34] in primates. This is consistent with imaging work showing that TMS delivered to motor cortex produced a strong activation in ipsilateral premotor cortex and other motor areas, but not motor cortex itself [35], [36]. As indicated by modeling work, this may be because both excitatory and inhibitory neurons are strongly activated at the site of stimulation [37], whereas in connected areas, such as premotor cortex, neurons are activated through long-range excitatory pathways, yielding larger responses and therefore a higher likelihood of potentiation (Esser et al., SLEEP Abstr. 0082, 2006).

### Synaptic plasticity and sleep SWA

As mentioned in the Introduction, SWA is a recognized marker of sleep need, but the mechanisms responsible for the homeostatic increase in SWA during wakefulness and its decrease during sleep remain unknown [3], [5]. The main goal of the present study was to test the hypothesis that sleep need may be linked to synaptic plasticity, by investigating whether the explicit induction of synaptic potentiation in localized cortical regions would increase the local level of sleep SWA [9], [38]. Using hd-EEG recordings during sleep, we found that when subjects went to sleep after 5 Hz rTMS, as opposed to after sham stimulation, they showed indeed a localized increase in SWA. Moreover, the increase in SWA was localized to the same cortical region–left premotor cortex–where 5 Hz rTMS had led to an increase in TMS-evoked potentials, indicating that the two effects were spatially specific and topographically congruent. Finally, the increase in TMS-evoked potentials during wakefulness predicted the magnitude of the increase in SWA during sleep, consistent with the possibility that the two effects may be causally linked. Thus, the present findings are consistent with the hypothesized link between synaptic strength and sleep SWA. It should be emphasized, however, that rTMS may cause changes in membrane excitability outside of synapses and shifts in the ratio between excitation and inhibition that may also contribute to the observed effects [39]. Moreover, the application of rTMS may also result in local changes in neuromodulation that could affect subsequent responses to test stimuli. Finally, it is important to note that the changes we observed over premotor cortex in terms both of increased TMS evoked responses during wakefulness and increases in local slow wave activity during sleep could be due to changes in synaptic efficacy or in membrane excitability not in the underlying circuits, but rather in connected brain regions that may even lie at considerable distance from the targeted structure. These include not only connected cortical areas such as somatosensory areas, supplementary motor area and certain parietal areas, but also subcortical structures, such as thalamic and spinal circuits that provide reafferents to the cortex.

While rTMS-induced potentiation of cortical evoked responses represents an effective method for replicating classic LTP paradigms in humans and to investigate the consequences on SWA homeostasis, its effects on cortical circuits are not physiological, since the hypersynchronous discharge of a majority of synapses is likely to produce a non-selective increase in synaptic strength. In this context, the results of previous studies offer a complementary view on the relationship between cortical plasticity and sleep SWA. Specifically, a recent hd-EEG study showed that a visuomotor learning task involving right parietal cortex [40] also led to a local increase in SWA during subsequent sleep, while a kinematically equivalent motor task not requiring learning did not [6]. Intriguingly, post-sleep task performance also improved, and the improvement was positively correlated with the local increase of SWA. Unlike the present TMS study, the learning study could not provide direct electrophysiological evidence of potentiation of local synaptic circuits. On the other hand, the learning study produced a cortical activation that was undoubtedly physiological. It is crucial, therefore, that a local increase in SWA was observed in both studies. Moreover, in both studies the increase in SWA was limited to the EEG frequencies known to be homeostatically regulated in physiological sleep [3]. Finally, the level of SWA decreased over time in both studies, in agreement with the homeostatic decline of SWA within and across sleep cycles [3].

Further evidence documenting a connection between synaptic plasticity and SWA regulation is provided by a recent study of arm immobilization [8]. In this study, after 12 hours of left arm immobilization during the day, motor performance deteriorated, and both somatosensory and motor evoked potentials decreased over contralateral sensorimotor cortex, indicative of local synaptic depression. During subsequent sleep, SWA over the same cortical area was also reduced, to an extent predicted by the decrease in performance. Mechanistically, the increase in SWA with synaptic potentiation and its decrease with synaptic depression appears to be due to changes in the dynamics of cellular slow oscillations and in the efficacy by which corticocortical synapses recruit and synchronize large populations of neurons (Hill and Tononi, SLEEP Abstr. 0011, 2006). Altogether, these studies are consistent with the notion that SWA, and presumably sleep need, are increased by events leading to synaptic potentiation and decreased by events leading to synaptic depression, and that their regulation can occur locally in cortical circuits [9], [38].

## Materials and Methods

Ten healthy right-handed male subjects (mean age 26.5±1.6 years) gave informed consent to participate in the study, which was approved by the local ethics committee. A neurological screening was performed to exclude subjects with conditions that could predispose them to potential adverse effects of TMS.

### TMS and sham TMS

TMS was targeted to the hand area of the left motor cortex throughout the experiment. The angulated coil was placed tangentially to the scalp with the handle pointing backwards. Precision and reproducibility of the stimulation were achieved by means of a Navigated Brain Stimulation (NBS) system (Nexstim Ltd). The NBS device uses an optical tracking system to locate the TMS coil relative to the subject's co-registered MR image and allows a digitization of the location of the EEG electrodes. A Magstim standard rapid rate stimulator with an air-cooled figure of eight coil was used for stimulation. Stimulus intensity was set relative to resting motor threshold (RMT), which was identified in the relaxed first dorsal interosseous of the right hand, where motor evoked potentials were recorded. The pre-and post-rTMS test phases consisted of 200 TMS pulses delivered every 0.5–0.7 seconds at 90% RMT. The conditioning phase consisted of 1500 TMS pulses delivered at 90% RMT. Repetitive TMS (rTMS) pulses were delivered at a base frequency of 5 Hz with pauses in stimulation determined according to safety guidelines [12], [41] (for a schematic representation of the stimulation paradigm see Supplemental Figure S2). The stimulation lasted for about 11 minutes. For safety purposes, subjects EEG was carefully monitored online during the TMS sessions. We observed no epileptiform EEG abnormalities. Furthermore, subjects were interviewed immediately following and one week after the experiment. Subjects reported no adverse effects. Each subject underwent one rTMS session and, at least one week earlier or later, a control experiment, where the same procedure was applied except that sham rTMS was delivered in the conditioning phase. For sham rTMS, the coil was rotated 90° around the axis of the handle and separated from the head using a 2 cm plastic spacer cube to ensure an indirect contact between the coil and the subjects head. The order of the experiments was randomized to control for order effect. At debriefing at the end of the experiment subjects did not report any difference between the two conditions. Offline, for the analysis of evoked responses, the data was average referenced, baseline corrected (100 ms prestimulus), band pass filtered (5–100 Hz) and averaged for each subject. Total EEG activity was assessed using the global mean field power (GMFP) [12], [42].

### TMS-evoked EEG responses

EEG responses to TMS were recorded by means of a cap with 60 carbon electrodes and a specifically designed TMS-compatible amplifier (Nexstim Ltd). The EEG signals were filtered (0.1–500 Hz) and sampled at 1450 Hz (for detail see [27]). Confounding factors such as auditory evoked responses and attentional effects on evoked responses were reduced by noise masking and by engaging the subject in a simple oddball task. In this task, interspersed within the noise masking, tones were played at irregular intervals (10–60 s) and the subject had to respond as fast as possible with a mouse button click with the left hand. Differences were assessed by paired t-tests.

### Sleep EEG recordings

After the TMS/EEG portion of the experiment the room was darkened and subjects were allowed to sleep in a bed. We then recorded the first sleep episode. Due to technical limitations of the available hd-EEG electrodes, it was not possible to record the full night of sleep. The sleep recording was therefore terminated at the first occurrence of REM sleep or when the subject woke up. Sleep EEG recordings for the first sleep episode were band-pass filtered between 0.1 and 40 Hz, downsampled to 128 Hz, and average-referenced. Sleep stages were visually scored for 20-s epochs according to standard criteria [43]. For a qualitative analysis of the sleep EEG, spectral analysis of consecutive 20-s epochs was performed for all 60 channels (FFT routine, Hanning window, averages of five 4-s epochs). Visual and semi-automatic artifact removal were performed [44]. Significant topographical differences in hd-EEG power during the first 30 min NREM sleep were assessed by statistical nonparametric mapping (SnPM) [6], [8], [45]. This method takes advantage of the actual data distribution and accounts for multiple comparisons testing in hd-EEG recordings. Briefly, EEG readings at each electrode for the potentiation condition and the control conditions were shuffled according to all possible permutations for all subjects. Based on the statistics obtained from the permutation data, we calculated a t-value for each electrode, and found the maximal t-value over all electrodes. The t-value threshold was taken as the 95th percentile of the permutation-derived t-values, and electrodes exceeding that threshold were taken as showing a significant difference between the two conditions. T-values presented in the figures are based on paired t-tests.

### Source localization

Source localization was performed on the average pre-conditioning TMS-evoked EEG response using the Curry software package (Curry 5.0, Neuroscan). Electrode positions were digitized and co-registered to each subject's MRI by means of an infrared positioning system (Nexstim). We then estimated the current density on the cortical surface by using the sLORETA algorithm [46]. The current density of the average evoked response was then projected onto the Montréal Neurological Institute (MNI) standard brain.

## Supporting Information

Figure S1 A. Topographic distribution of power in the frequency ranges significantly affected by 5-Hz conditioning illustrated in Figure 3A. Average EEG power density (n = 10 subjects) for the first 30 minutes of NREM sleep after the 5-Hz conditioning (top), the sham control condition (middle), and the relative change between the two (bottom). Values were normalized by total power for the recording, color coded, plotted at the corresponding position on the planar projection of the scalp surface, and interpolated (biharmonic spline) between electrodes (dots). Values to the left of the topographic plots represent maximal and minimal power (in percentage of the overall mean) with standard errors in parenthesis. White dots indicate electrodes showing significant differences after statistical non-parametric mapping (see methods). B. Time course of changes in power in the respective frequency ranges after 5-Hz conditioning in 10-min intervals. We selected power at electrode 9, corresponding to the peak SWA increase.
(1.06 MB PDF)

Figure S2 Schematic representation of the rTMS stimulation paradigm. Pulses were organized into bursts of 50 pulses delivered at 5 Hz. Bursts were organized into trains of six bursts, with each separated by 5 s. A total of five trains were delivered, each separated by 1 min. The stimulation paradigm was adapted from studies reporting long-lasting changes of motor evoked potential after such rTMS conditioning (Peinemann et al., 2004 and Quartarone et al., 2005).
(0.17 MB PDF)
