# Supplementary figures and images for: TMS-Induced Cortical Potentiation during Wakefulness Locally Increases Slow Wave Activity during Sleep
Source: PLoS One. 2007 Mar 7;2(3):e276. doi: 10.1371/journal.pone.0000276 (PMC1803030; doi:10.1371/journal.pone.0000276)

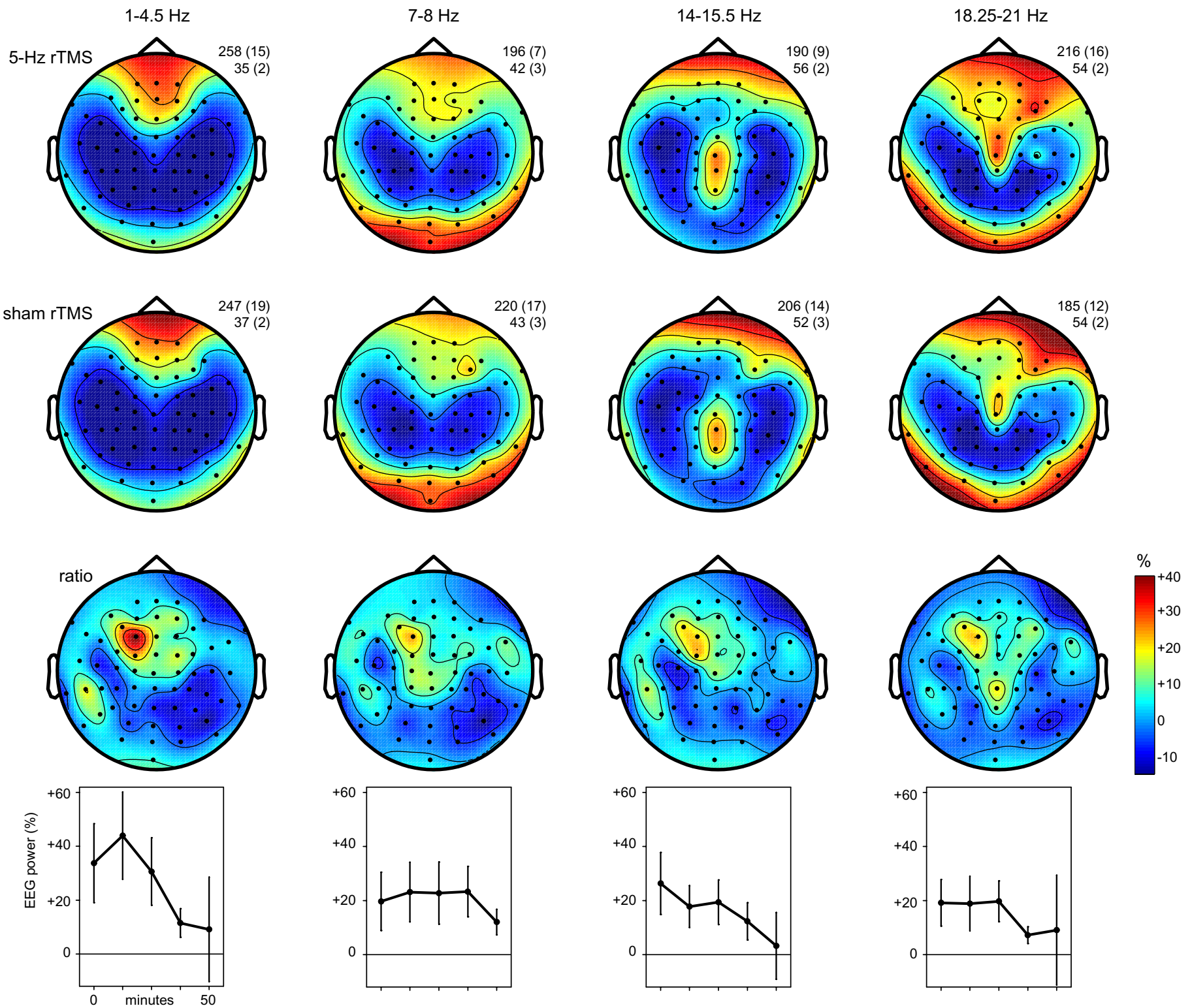

Supplement: Figure S1 — A. Topographic distribution of power in the frequency ranges significantly affected by 5-Hz conditioning illustrated in Figure 3A. Average EEG power density (n = 10 subjects) for the first 30 minutes of NREM sleep after the 5-Hz conditioning (top), the sham control condition (middle), and the relative change between the two (bottom). Values were normalized by total power for the recording, color coded, plotted at the corresponding position on the planar projection of the scalp surface, and interpolated (biharmonic spline) between electrodes (dots). Values to the left of the topographic plots represent maximal and minimal power (in percentage of the overall mean) with standard errors in parenthesis. White dots indicate electrodes showing significant differences after statistical non-parametric mapping (see methods). B. Time course of changes in power in the respective frequency ranges after 5-Hz conditioning in 10-min intervals. We selected power at electrode 9, corresponding to the peak SWA increase. (1.06 MB PDF) [file pone.0000276.s001.pdf]

### 5-Hz rTMS conditioning stimulation paradigm

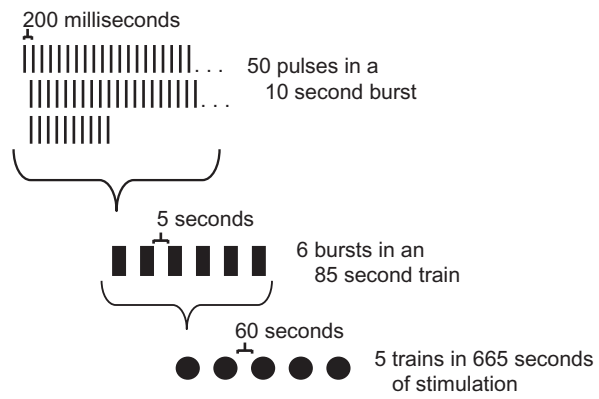

Supplement: Figure S2 — Schematic representation of the rTMS stimulation paradigm. Pulses were organized into bursts of 50 pulses delivered at 5 Hz. Bursts were organized into trains of six bursts, with each separated by 5 s. A total of five trains were delivered, each separated by 1 min. The stimulation paradigm was adapted from studies reporting long-lasting changes of motor evoked potential after such rTMS conditioning (Peinemann et al., 2004 and Quartarone et al., 2005). (0.17 MB PDF) [file pone.0000276.s002.pdf]
